# Supplementary material for: Anti-racist and anti-colonial content within US global health curricula
Source: PLOS Glob Public Health. 2025 Feb 6;5(2):e0003710. doi: 10.1371/journal.pgph.0003710 (PMC11801725; doi:10.1371/journal.pgph.0003710)
Supplement: S1 Questionnaire — (PDF) [file pgph.0003710.s001.pdf]

Anti-racism and anti-colonialism content within US global health curricula

Please complete the survey below.

Thank you!

This survey is designed to assess the current landscape of global health education in the areas of anti-racism and decolonization and inform the development of a curriculum aimed at preparing learners to have mutually beneficial, cross-cultural partnerships.

It has been IRB-approved by the Baylor College of Medicine Institutional Review Boards. Participation in the survey implies consent to participate in the study. This survey should be completed by the global health program director or the faculty member most familiar with your global health program's experiences / education. Please note that we would prefer to have one survey respondent per institution. If you represent both a global health residency and fellowship we ask that you complete the survey twice so data may be analyzed correctly.

Time to complete the survey will vary depending on the depth of global health opportunities offered in your program and will take anywhere from 5 to 30 minutes to complete. We would be very grateful for your participation in this survey and look forward to sharing the results with you.

If you would like someone else to complete the survey in your place, please forward this email to the appropriate person.

Consent statement (click box): I hereby acknowledge that I have read the introduction and choose to voluntarily participate in this survey.	☐ Agree

Program description

Please answer the following questions about the global health program you represent and your role as a global health educator.

The program I represent is located in the:	◯ Mid-America region: Indiana, Kentucky, Michigan, Ohio, West Pennsylvania, West Virginia<br>◯ Mid-Atlantic region: Delaware, East Pennsylvania, Maryland, Southern New Jersey, Washington D.C.<br>◯ Midwest region: Illinois, Iowa, Kansas, Minnesota, Missouri, Nebraska, Oklahoma, South Dakota, Wisconsin<br>◯ New York region: New York, Northern New Jersey<br>◯ Northeast region: Connecticut, Maine, Massachusetts, New Hampshire, Rhode Island, Vermont<br>◯ Southeast region: Alabama, Arkansas, Florida, Georgia, Louisiana, Mississippi, North Carolina, South Carolina, Tennessee, Virginia<br>◯ Southwest region:Texas<br>◯ Western region: Alaska, Arizona, California, Colorado, Hawaii, Nevada, New Mexico, Oregon, Utah, Washington<br>◯ None of the above
Where is the program you represent located?	__________________________________
The program I represent is affiliated with the military.	◯ Yes<br>◯ No
The program I represent is affiliated with a university or academic center.	◯ Yes<br>◯ No
I represent a:	◯ Global Health Residency program or track<br>◯ Global Health Fellowship program or track<br>◯ Other
What other type of global health program do you represent?	__________________________________
What residency specialty do you represent?	◯ Emergency Medicine<br>◯ Family Medicine<br>◯ Global/International Emergency Medicine Residency<br>◯ Internal Medicine<br>◯ Internal Medicine/Pediatrics<br>◯ Pediatrics<br>◯ Other
Please describe your other specialty:	__________________________________

What specialty is the global health fellowship you represent associated with?

- ◯ Emergency Medicine Global Health
- ◯ Family Medicine Global Health
- ◯ Pediatric Global Health
- ◯ Global Health
- ◯ Internal Medicine Global Health
- ◯ Pediatric Cardiology
- ◯ Pediatric Critical Care Medicine
- ◯ Pediatric Emergency Medicine
- ◯ Pediatric Endocrinology
- ◯ Pediatric Gastroenterology
- ◯ Pediatric Hematology-Oncology
- ◯ Pediatric Infectious Diseases
- ◯ Pediatric Nephrology
- ◯ Pediatric Pulmonology
- ◯ Pediatric Rheumatology
- ◯ Other

Please describe your other specialty:

____

What is your position?

- ◯ Global Health Residency Program Director/Assistant Director
- ◯ Global Health Residency Track Director/Assistant Director
- ◯ Global Health Educator
- ◯ Other

Please specify your other position:

____

What is your position?

- ◯ Global Health Fellowship Program Director/Assistant Director
- ◯ Global Health Fellowship Track Director/Assistant Director
- ◯ Global Health Educator
- ◯ Other

Please specify your other position:

____

Which of the following does your program offer (select all that apply):

- ☐ GH residency with over 3 months of training outside the USA
- ☐ Short term GH (< 3 months outside the USA) elective for residents
- ☐ Short term (< 3 months) domestic border health rotation
- ☐ Long term (>3 months) domestic border health rotation
- ☐ Short term (< 3 months) Indian Health Services rotation
- ☐ Long term (>3 months) Indian Health Services rotation
- ☐ Other

Please describe other:

____

Which of the following does your program offer (select all that apply):	☐ GH fellowship with over 3 months of training outside the USA<br>☐ Short term GH (< 3 months outside the USA) elective for fellows<br>☐ Short term (< 3 months) domestic border health rotation<br>☐ Long term (>3 months) domestic border health rotation<br>☐ Short term (< 3 months) Indian Health Services rotation<br>☐ Long term (>3 months) Indian Health Services rotation<br>☐ Other
Please describe other:	__________________________________

What year did the first class of your global health program start?

- ◯ I don't know ◯ Before 1980
- ◯ 1980 ◯ 1981 ◯ 1982
- ◯ 1983 ◯ 1984 ◯ 1985
- ◯ 1986 ◯ 1987 ◯ 1988
- ◯ 1989 ◯ 1990 ◯ 1991
- ◯ 1992 ◯ 1993 ◯ 1994
- ◯ 1995 ◯ 1996 ◯ 1997
- ◯ 1998 ◯ 1999 ◯ 2000
- ◯ 2001 ◯ 2002 ◯ 2003
- ◯ 2004 ◯ 2005 ◯ 2006
- ◯ 2007 ◯ 2008 ◯ 2009
- ◯ 2010 ◯ 2011 ◯ 2012
- ◯ 2013 ◯ 2014 ◯ 2015
- ◯ 2016 ◯ 2017 ◯ 2018
- ◯ 2019 ◯ 2020 ◯ 2021

How many trainees does your global health residency program or track have per year?

- ◯ I don't know
- ◯ 0
- ◯ 1
- ◯ 2
- ◯ 3
- ◯ 4
- ◯ 5
- ◯ 6
- ◯ 7
- ◯ 8
- ◯ 9
- ◯ 10
- ◯ 11
- ◯ 12
- ◯ 13
- ◯ 14
- ◯ 15
- ◯ 16
- ◯ 17
- ◯ 18
- ◯ 19
- ◯ 20
- ◯ Over 20

How many trainees does your global health fellowship program or track have per year?

- ◯ I don't know
- ◯ 0
- ◯ 1
- ◯ 2
- ◯ 3
- ◯ 4
- ◯ 5
- ◯ 6
- ◯ 7
- ◯ 8
- ◯ 9
- ◯ 10
- ◯ 11
- ◯ 12
- ◯ 13
- ◯ 14
- ◯ 15
- ◯ 16
- ◯ 17
- ◯ 18
- ◯ 19
- ◯ 20
- ◯ Over 20

In which countries do your trainees most frequently participate in global health experiences?

☐ N/A
☐ Afghanistan
☐ Albania
☐ Algeria
☐ Andorra
☐ Angola
☐ Antigua and Barbuda
☐ Argentina
☐ Armenia
☐ Australia
☐ Austria
☐ Azerbaijan
☐ The Bahamas
☐ Bahrain
☐ Bangladesh
☐ Barbados
☐ Belarus
☐ Belgium
☐ Belize
☐ Benin
☐ Bhutan
☐ Bolivia
☐ Bosnia and Herzegovina
☐ Botswana
☐ Brazil
☐ Brunei
☐ Bulgaria
☐ Burkina Faso
☐ Burundi
☐ Cambodia
☐ Cameroon
☐ Canada
☐ Cape Verde
☐ Central African Republic
☐ Chad
☐ Chile
☐ China
☐ Colombia
☐ Comoros
☐ Congo (Republic of the)
☐ Congo (Democratic Republic of the)
☐ Costa Rica
☐ Cote d'Ivoire
☐ Croatia
☐ Cuba
☐ Cyprus
☐ Czech Republic
☐ Denmark
☐ Djibouti
☐ Dominica
☐ Dominican Republic
☐ East Timor (Timor-Leste)
☐ Ecuador
☐ Egypt
☐ El Salvador
☐ Equatorial Guinea
☐ Eritrea
☐ Estonia
☐ Ethiopia
☐ Fiji
☐ Finland
☐ France
☐ Gabon
☐ The Gambia
☐ Georgia
☐ Germany
☐ Ghana
☐ Greece
☐ Grenada

- ☐ Guatemala
- ☐ Guinea
- ☐ Guinea-Bissau
- ☐ Guyana
- ☐ Haiti
- ☐ Honduras
- ☐ Hungary
- ☐ Iceland
- ☐ India
- ☐ Indonesia
- ☐ Iran
- ☐ Iraq
- ☐ Ireland
- ☐ Israel
- ☐ Italy
- ☐ Jamaica
- ☐ Japan
- ☐ Jordan
- ☐ Kazakhstan
- ☐ Kenya
- ☐ Kiribati
- ☐ North Korea
- ☐ South Korea
- ☐ Kosovo
- ☐ Kuwait
- ☐ Kyrgyzstan
- ☐ Laos
- ☐ Latvia
- ☐ Lebanon
- ☐ Lesotho
- ☐ Liberia
- ☐ Libya
- ☐ Liechtenstein
- ☐ Lithuania
- ☐ Luxembourg
- ☐ Macedonia
- ☐ Madagascar
- ☐ Malawi
- ☐ Malaysia
- ☐ Maldives
- ☐ Mali
- ☐ Malta
- ☐ Marshall Islands
- ☐ Mauritania
- ☐ Mauritius
- ☐ Mexico
- ☐ Micronesia (Federated States of)
- ☐ Moldova
- ☐ Monaco
- ☐ Mongolia
- ☐ Montenegro
- ☐ Morocco
- ☐ Mozambique
- ☐ Myanmar (Burma)
- ☐ Namibia
- ☐ Nauru
- ☐ Nepal
- ☐ Netherlands
- ☐ New Zealand
- ☐ Nicaragua
- ☐ Niger
- ☐ Nigeria
- ☐ Norway
- ☐ Oman
- ☐ Pakistan
- ☐ Palau
- ☐ Panama
- ☐ Papua New Guinea
- ☐ Paraguay
- ☐ Peru
- ☐ Philippines

- [ ] Poland
- [ ] Portugal
- [ ] Qatar
- [ ] Romania
- [ ] Russia
- [ ] Rwanda
- [ ] Saint Kitts and Nevis
- [ ] Saint Lucia
- [ ] Saint Vincent and the Grenadines
- [ ] Samoa
- [ ] San Marino
- [ ] Sao Tome and Principe
- [ ] Saudi Arabia
- [ ] Senegal
- [ ] Serbia
- [ ] Seychelles
- [ ] Sierra Leone
- [ ] Singapore
- [ ] Slovakia
- [ ] Slovenia
- [ ] Solomon Islands
- [ ] Somalia
- [ ] South Africa
- [ ] South Sudan
- [ ] Spain
- [ ] Sri Lanka
- [ ] Sudan
- [ ] Suriname
- [ ] Swaziland
- [ ] Sweden
- [ ] Switzerland
- [ ] Syria
- [ ] Taiwan
- [ ] Tajikistan
- [ ] Tanzania
- [ ] Thailand
- [ ] Togo
- [ ] Tonga
- [ ] Trinidad and Tobago
- [ ] Tunisia
- [ ] Turkey
- [ ] Turkmenistan
- [ ] Tuvalu
- [ ] Uganda
- [ ] Ukraine
- [ ] United Arab Emirates
- [ ] United Kingdom
- [ ] United States of America
- [ ] Uruguay
- [ ] Uzbekistan
- [ ] Vanuatu
- [ ] Vatican City (Holy See)
- [ ] Venezuela
- [ ] Vietnam
- [ ] Yemen
- [ ] Zambia
- [ ] Zimbabwe
- [ ] Other

Please indicate in which other country (countries) your trainees participate in global health experiences.

____

Please indicate where in the United States your trainees participate in global health experiences.

____

Does your US-based global health program have faculty educators from the following groups? (select all that apply):

- ☐ Medical practitioners from a historically underrepresented minority group in medicine (URM) (Black/African American, Latinx, American Indians, Alaska Natives, and Native Hawaiians)
- ☐ Medical practitioners who are international medical graduates (IMGs)
- ☐ None of the above
- ☐ I don't know

Please provide your global health residency or fellowship program link here (optional):

____

May we contact you if we need further clarification on any of your responses?

- ◯ Yes
- ◯ No

Preferred method(s) of contact:

- ☐ Email
- ☐ Phone

Email address

____

Phone number:

____

Global Health Curriculum Content

The following section contains questions about whether your program's global health education curriculum provides training in the areas of colonial history, anti-racism and cultural sensitivity.

Does your program require training utilizing a formal pre-departure preparation curriculum prior to international rotations? (select one)

- ◯ Yes
- ◯ No
- ◯ I don't know

Does your program require training utilizing a formal pre-departure preparation curriculum prior to domestic underserved site rotations? (select one)

- ◯ Yes
- ◯ No
- ◯ I don't know

Are trainees at your program required to learn about the following as part of their international pre-departure training? (select all that apply)

- ☐ Host country history and geo-politics
- ☐ Host country culture
- ☐ Host country language
- ☐ None of the above
- ☐ Not applicable
- ☐ I don't know

Why are trainees not required to learn about the above topics as part of their international pre-departure training?

____

Are trainees at your program required to learn about the following as part of their domestic underserved rotation pre-departure training? (select all that apply)

- ☐ Host site local history and geo-politics
- ☐ Host site culture
- ☐ Host site language
- ☐ None of the above
- ☐ Not applicable
- ☐ I don't know

Why are trainees at your program not required to learn about the above topics as part of their domestic underserved rotation pre-departure training?

____

Are global health program faculty who travel internationally required to learn about the following as part of their pre-departure training? (select all that apply)

- ☐ Host country history and geo-politics
- ☐ Host country culture
- ☐ Host country language
- ☐ None of the above
- ☐ I don't know

Why are faculty at your program not required to learn about the above topics as part of their international pre-departure training?

____

Are global health program faculty who engage in work at domestic underserved sites required to learn about the following as part of their pre-departure training? (select all that apply)

- ☐ Host site local history and geo-politics
- ☐ Host site culture
- ☐ Host site language
- ☐ None of the above
- ☐ I don't know

Why are faculty at your program not required to learn about the above topics as part of their domestic underserved rotation pre-departure training?

____

Which of the following topics does your global health education curriculum address?

	Not covered	Minimal exposure (mentioned in at least one lecture or activity, not a learning objective)	Moderately covered (a specific learning objective in at least one lecture or activity)	Strongly emphasized (a specific learning objective in two different lectures or activities, a research activity or written assignment topic)	I don't know
History of global health	○	○	○	○	○
History of colonialism	○	○	○	○	○
History of racial health disparities in the United States	○	○	○	○	○
Current racial health disparities in the United States	○	○	○	○	○
Anti-racism (defined as policies and practices that oppose racism and promote racial tolerance)	○	○	○	○	○

Indigenous health	○	○	○	○	○
Immigrant and refugee health	○	○	○	○	○
"White saviorism" (defined as concept by which a white person or person from a privileged background acts to help non-white or disadvantaged people, but in a context which can be perceived as self-serving)	○	○	○	○	○
Privilege and power dynamics between formerly colonizing and formerly colonized countries	○	○	○	○	○
Critical consciousness (defined as a level of sociopolitical awareness through which a person understands their positionality in the world)	○	○	○	○	○
Examination of motivation for global health engagement	○	○	○	○	○
Cultural humility (defined by Hook et al., 2013 as "the ability to maintain an interpersonal stance that is other-oriented (or open to the other) in relation to aspects of cultural identity that are most important to the [person]")	○	○	○	○	○
Health equity/inequity	○	○	○	○	○
Social media practices	○	○	○	○	○
Photography ethics	○	○	○	○	○

Are there any other educational components in the areas of equity, anti-racism and decolonization in your global health program that you would like to list or describe? __________________________________

How are the following topics taught in your global health curriculum? (select all that apply):

	Not covered	Didactic lecture (remote or in person)	Workshop / role play/ simulation	Case or journal club discussion s	Online module	Self-direct ed/ readings	Other	I don't know
History of global health	☐	☐	☐	☐	☐	☐	☐	☐
History of colonialism	☐	☐	☐	☐	☐	☐	☐	☐

History of racial health disparities in the United States	☐	☐	☐	☐	☐	☐	☐	☐
Current racial health disparities in the United States	☐	☐	☐	☐	☐	☐	☐	☐
Anti-racism (defined as policies and practices that oppose racism and promote racial tolerance)	☐	☐	☐	☐	☐	☐	☐	☐
Indigenous health	☐	☐	☐	☐	☐	☐	☐	☐
Immigrant and refugee health	☐	☐	☐	☐	☐	☐	☐	☐
"White saviorism" (defined as a concept by which a white person or person from a privileged background acts to help non-white or disadvantaged people, but in a context which can be perceived as self-serving)	☐	☐	☐	☐	☐	☐	☐	☐
Privilege and power dynamics between formerly colonizing and formerly colonized countries	☐	☐	☐	☐	☐	☐	☐	☐
Critical consciousness (defined as a level of sociopolitical awareness through which a person understands their positionality in the world)	☐	☐	☐	☐	☐	☐	☐	☐
Examination of motivation for global health engagement	☐	☐	☐	☐	☐	☐	☐	☐
Cultural humility (defined by Hook et al., 2013 as "the ability to maintain an interpersonal stance that is other-oriented (or open to the other) in relation to aspects of cultural identity that are most important to the [person]")	☐	☐	☐	☐	☐	☐	☐	☐
Health equity/inequity	☐	☐	☐	☐	☐	☐	☐	☐
Social media practices	☐	☐	☐	☐	☐	☐	☐	☐
Photography ethics	☐	☐	☐	☐	☐	☐	☐	☐

Please describe other teaching methods your program employs to cover the above topics: ______________________________

Does your program have written policies addressing the following topics?

	Yes	No	I don't know
Social media posts while on global health rotations	○	○	○
Photography ethics while on global health rotations	○	○	○
Trainee clinical scope of practice while on global health rotations	○	○	○

Research

The following section contains questions about your trainees' research experience.

Do your trainees participate in research/scholarship projects during their global health rotation?

- ○ Research is elective
- ○ Research is required
- ○ No
- ○ I don't know

Are trainees required to engage host site collaborators as co-authors/co-investigators on research or scholarship projects?

- ○ They are required
- ○ They are encouraged but not required
- ○ They are neither required nor encouraged
- ○ I don't know

Who sets the research objectives?

- ○ Your program/institution
- ○ Your host institution
- ○ Both
- ○ Other
- ○ I don't know

Please describe other:

____

Please describe challenges (if applicable) encountered by your program in having your collaborators participate in setting research objectives.

____

Are trainees paired with a host site mentor (local faculty or clinician who assists in research design and execution) for research?

- ○ All of the time
- ○ Sometimes
- ○ Never
- ○ I don't know

Please describe challenges (if applicable) encountered by your program in pairing trainees with host site research mentors.

____

Partnership Equity

The following section contains questions about your global health program's partnership practices.

Prior to the COVID-19 pandemic, did your program host trainees from low and middle income countries (LMICs)?

- ◯ Yes
- ◯ No
- ◯ I don't know

Please describe how the rotation is arranged, funded and what activities your guests from LMICs participate in while visiting your institution in the USA:

____

On average, in an academic year pre-COVID era, how many trainees from your program in the US visited your partner site(s) in an LMIC?

- ◯ 0
- ◯ 1
- ◯ 2
- ◯ 3
- ◯ 4
- ◯ 5
- ◯ 6
- ◯ 7
- ◯ 8
- ◯ 9
- ◯ 10
- ◯ 11
- ◯ 12
- ◯ 13
- ◯ 14
- ◯ 15
- ◯ 16
- ◯ 17
- ◯ 18
- ◯ 19
- ◯ 20
- ◯ Over 20
- ◯ I don't know

On average, in an academic year pre-COVID era, how many trainees from your partner LMIC site(s) visited your program in the US?

- ◯ 0
- ◯ 1
- ◯ 2
- ◯ 3
- ◯ 4
- ◯ 5
- ◯ 6
- ◯ 7
- ◯ 8
- ◯ 9
- ◯ 10
- ◯ 11
- ◯ 12
- ◯ 13
- ◯ 14
- ◯ 15
- ◯ 16
- ◯ 17
- ◯ 18
- ◯ 19
- ◯ 20
- ◯ Over 20
- ◯ I don't know

Please describe what challenges your program has faced in attempting to host trainees from your LMIC partner site(s):

____

Are trainee evaluations by the host site required at your program?

- ◯ Yes
- ◯ No
- ◯ I don't know

Do your host site preceptors/mentors formally assess trainees in clinical settings on:

	Yes	No	Not applicable	I don't know
Cultural humility	◯	◯	◯	◯
Cross cultural communication	◯	◯	◯	◯
Ability to adapt to local setting	◯	◯	◯	◯
Ability to integrate local team	◯	◯	◯	◯

Do your host site preceptors/mentors formally assess trainees in research settings on:

	Yes	No	Not applicable	I don't know
Cultural humility	◯	◯	◯	◯
Cross cultural communication	◯	◯	◯	◯
Ability to adapt to local setting	◯	◯	◯	◯
Ability to integrate local team	◯	◯	◯	◯

Do your host site preceptors/mentors have access to a formal mechanism to report concerns regarding inappropriate practices or behavior by visiting trainees?

- ◯ Yes
- ◯ No
- ◯ I don't know

Without disclosing identifying information, please describe instances of concerns regarding inappropriate practices or behavior by visiting trainees raised by your partners.

____

Final thoughts

What tools do you think would be most helpful to your program to integrate the topics discussed above into your global health curricula?

Please add any other comments you would like to share with us.
