# Supplementary material for: Anti-racist and anti-colonial content within US global health curricula
Source: PLOS Glob Public Health. 2025 Feb 6;5(2):e0003710. doi: 10.1371/journal.pgph.0003710 (PMC11801725; doi:10.1371/journal.pgph.0003710)
Supplement: S1 Appendix — (DOCX) [file pgph.0003710.s002.docx]

**S1 Appendix**

**Author Positionality Statements**

**S. Aya Fanny**

I am a black female born in Cote d’Ivoire who immigrated to the United States during adolescence and became an American citizen by naturalization. As such, I consider myself an immigrant and citizen of a formerly colonized nation, while acknowledging that I now enjoy the privileges that come with American citizenship and practicing medicine in the United States of America. When I am in the United States, I navigate my daily life as a minority. However, I am aware that during my global health engagements abroad, I am seen as an American doctor and that my interactions with others are inherently susceptible to power imbalances.

**Amy Rule**

I am a white, disabled female born in the United States. My race and citizenship have offered considerable privileges throughout my life. As a professional I have extraordinary privilege as a global health education leader through the Association of Pediatric Program Directors and the American Academy of Pediatrics, as well as a global health education leader at my home institution. In my global health maternal-newborn research in East Africa, I am aware of the privilege I experience as a white, American doctor and that my interactions with my colleagues there are subject to power imbalances of both present and historic structural inequity and bias.  My interest in global health ethics and anti-colonialism and anti-racism practice in global health grew from the contrast of my initial experiences in global health working with disability rights group in Eastern Europe as a disabled person and working in East Africa as a white American. In the Eastern European context, I was often mistaken for as unhoused individual and/or patients because of my visible disability and the stigma that carried. In contrast, in East Africa as a visiting white American trainee, I was offered numerous privileges over my East African peers.  These contrasting experiences early in my career and training sparked my interest and commitment to critical reflection on global health engagement and assuring that such reflection becomes a standard part of global health training.

**Heather L. Crouse**

I am a U.S.-born, white female raised in the Southern U.S. and privileged to have lived, studied and worked in many countries around the world. I recognize that I benefit from majority status in my U.S. work as a physician and white privilege in international settings.

**James Hudspeth**

I am a US-born white male with two physician parents. I acknowledge the privilege my intersecting identities afford me both within the US and globally.

**Bethany Hodge**

I am a US-born, white female raised and practicing in rural Midwest America. I benefit from majority status in my US work and white privilege in international settings. Being cognizant of this, I have focused my global health on examining the true outcomes experienced by both the participants and recipients of our activities and the translation of good intentions into useful work.

**Marideth Rus**

I am a US-born, white female raised in Midwest America and practicing in the Southern US. I recognize that I benefit from white privilege as well as my status as a physician and US citizen.

**Heather Haq**

I am a U.S.-born, mixed-race female of white and Pakistani heritage, with both American and immigrant family members. As a white-presenting female, I benefit from white privilege as well as my status as a U.S. citizen and status as a physician.
