# Supplementary material for: Anti-racist and anti-colonial content within US global health curricula
Source: PLOS Glob Public Health. 2025 Feb 6;5(2):e0003710. doi: 10.1371/journal.pgph.0003710 (PMC11801725; doi:10.1371/journal.pgph.0003710)
Supplement: Attachment — Submitted filename: SAF ARAC curricula PLOS PGH Rebuttal Letter 6Nov24 .pdf [file pgph.0003710.s004.pdf]

Dear Editor and Reviewers:

We thank you for your thoughtful assessment of our work and the opportunity you have given us to address them and resubmit our manuscript. Please find below our detailed responses to your queries.

Editor's comments with responses:

1. The reviewer points out the lack of an LMIC gaze/pose or both. Even though the focus of the paper is on US curricula, the reviewer laments the disadvantage the authors have of the absence of an external view at these curricula. This can easily be couched as a limitation, even if we can argue that those taking a curriculum are in the best position to critique it.

We appreciate the reviewer's point on this and have added this as a limitation to our study; it is also commented on in our reflexivity statement. As noted above, while the focus is on US curricula being created and used by our study participants, having input during the course of the study from LMIC colleagues would have added depth to the process and potentially offered new insights.

2. Please move the group positionality statement into the main text of the study under methods- perhaps under a heading like "Researcher's Reflexivity". You can leave the individual statements in the supplements.

We have done this.

3. With regards to the ideal categorization of the longitudinal curriculum, and the curriculum in general, this is a retrospective look at what exists, and these categories have already been used in collecting data. Please discuss within limitations that more concise and clear categories could be used reflecting on the reviewer's suggestions.

We have added discussion of the categories to both the methods (detailing how the cited reference arrived at these categories) and to the limitations.

4. Also respond to the content query around global history and unpack whether this is contained in "history of global health (history of colonialism, history of global health), history of manifest destiny (indigenous health, history of US racial disparities, current US racial health disparities, immigrant and refugee health, health inequity).
Due to it's sensitivity, I would suggest that you replace the history of manifest destiny (which can be misinterpreted for endorsement), even if that was what was originally used, with more direct and clear terminology that perhaps calls out the challenge with the concept of "manifest destiny".

Comments on global history below. In terms of "manifest destiny," we appreciate the feedback and have changed the term to "US Health Disparities: Past & Present" in our figure and text. The term was not used in the initial survey.

5. Please make it clear in the data availability statement that Dr. Victor Gonzalez, director of research operations for the division of Pediatric Emergency Medicine at Baylor College of Medicine, at vmgonzal@bcm.edu will not be the primary/sole individual handling or determining the availability of this de-identified data. See Plos guidelines and please provide contact information for a data access committee, ethics committee, or other institutional body to which data requests may be sent. "When possible, we recommend authors deposit restricted data to a repository that allows for controlled data access. *If this is not possible, directing data requests to a non-author institutional point of contact, such as a data access or ethics committee, helps guarantee long term stability and availability of data. Providing interested researchers with a durable point of contact ensures data will be accessible even if an author changes email addresses, institutions, or becomes unavailable to answer requests." Please make the committee these requests are going to clear, and provide an institutional/committee email with "attention" contact to address these concerns.

The data has been uploaded to Dataverse, an open source data repository. It is available at: https://doi.org/10.15139/S3/X3E8A2.

Reviewer 2 comments:

1- The lack of LMIC authors is a major limitation in this study. This should be clearly listed as a major limitation in the discussion section of this study. The implications of this limitation should be thoroughly discussed.

   Addressed as a limitation as well as within the reflexivity statement.

2- It is good that the authors have uploaded their individual and group positionality statements. However, the group positionality statement needs to be included in the main text of the study under introduction or methods and not merely in the appendix.

   Our group reflexivity statement can now be found in the methods section under the heading “Researcher's reflexivity”.

3- Line 118 the term ‘history of manifest destiny’ is not the best term to use. It has colonial overtone.

   We welcome this point. We have changed this in the figure and text to “US Health Disparities: Past & Present”.

4- Would be better if the categories are:
   History of global health (history of colonialism, history of global health),
   Manifestations of inequities (indigenous health, history of US racial disparity, current US racial health disparities, immigrant and refugee health, health inequities)

Systemic bias (anti-racism, white saviourism, critical consciousness).
Intersectionality does not seem to belong here and isn't explicitly included in your curriculum.
Global health engagement (cultural humility, examination of motivation for GH engagement, social media, photography ethics)

We appreciate the perspective of the reviewer. The categories resulted from both literature review and a needs assessment of global health educators at a conference - we understand that there will be reasonable differences in perspective on how best to break down categories across scholars in our field, and worked to ensure these were not solely the opinions of our author group. We have added a comment about the limitations of these categories and expanded the discussion in methods as to how they were arrived at.

5- There should be more curriculum content on global history rather than merely US history. Why was this not included in the survey?

We agree with the reviewer that more curricular content on global history would be helpful. The survey was constructed with the insights of our authorship group, who collectively have reviewed a range of GME GH curricula, as well as the needs assessment discussed under point 4. Likely due to the limited time, few of curricula we know have expansive discussions of global history - most focus on the history of partner countries and the history of organizations that most directly impact global health (WHO, World Bank, etc). All curricula require triage of topics in the limited time available, and given that many of these only have 10-20 hours of contact time and a limited ability to assign readings given the rigorous clinical work of the trainees, we appreciate that their creators have to make challenging choices between the array of possible topics. For our categorizations, elements of "global history" would fall under the "history of global health" and "history of colonialism" sections.
