# Supplementary material for: Anti-racist and anti-colonial content within US global health curricula
Source: PLOS Glob Public Health. 2025 Feb 6;5(2):e0003710. doi: 10.1371/journal.pgph.0003710 (PMC11801725; doi:10.1371/journal.pgph.0003710)
Supplement: Attachment — Submitted filename: SAF PLOS GH Rebuttal letter 11Aug24 Final.docx [file pgph.0003710.s003.docx]

Dear Editor and Reviewers:

We thank you for your thorough and critical assessment of our work. We appreciate the opportunity you have given us to address them and resubmit our manuscript. Please find below our detailed responses to your queries.

1- Abstract: The survey was distributed to 148 GH programs. It is not clear if these were program leads? If there are 148 GH programs or these were 148 persons involved in GH? Please clarify because the survey was shared to persons. As is in methods, line 125 saying the 148 were educators is clearer.

Thank you for your suggestion. We have reworded the abstract to now read on pg 2, ln 55: *“The survey was distributed to 148 GH program educational leaders”*

2- Introduction; Not sure if the [ ] brackets in line 96 were intended. They don't seem serve any purpose.

Thank you for your suggestion. The brackets were to acknowledge that the original citation from Abimbola and Pai (2020) was truncated. We have removed them as suggested.

3- From the methods, it is not clear if this represents the whole US or these were respondents from a specific state/ geographical area in the US. Would be good in the methods to expound more on the study population. To whom the survey was shared and how it was shared.

The survey was distributed electronically (by email) to all programs located in the entirety of the United States that we identified through previous distribution lists and publicly available listings. We have added educational roles descriptions to the methods to be clearer on our survey recipients’ identity. Table 1 shows that all regions of the United States were represented in our study population. This is described now on pg 6 ln 132-134: *“Survey participants were identified through distribution lists from previous surveys of GH pediatric residencies(Butteris et al. 2015) and fellowships(Crouse et al. 2020) based in the United States of America, and publicly available listings of FM and EM GH tracks and fellowships.”*

4- remove the word sample in line 142.

This has been removed.

5- Discussion: Line 265 One would say that at least two thirds may represent diversity. The authors could acknowledge this and to maintain their argument probably justify why 100% would be the most ideal. Probably examine whether the host faculty would be involved with a pre-departure session virtually for trainees to hear from such faculty or the appointment of such faculty as visiting faculty in the US on the program.

The paragraph has been edited to incorporate your feedback on pg 13 ln 276-288.

6- Line 276 Could also be that the respondents to the survey were the group that is already converted and enthusiastic to see reforms and therefore would support activities/ surveys whose goal is to reform GH.

Thank you for this suggestion. We agree with you and have edited the text to reflect this consideration on pg 13 ln 292-294. “Our study’s ratio of 4:3 U.S. to LMIC learners is likely well above average, reflecting more advanced, better resourced programs that are likely to have already embraced ARAC principles; the dearth of systematic studies makes this hard to assess.”

7- Line 297/298 could come earlier in the paragraph to show how anti racism and anti colonialism were examined at institutional level so that the section is seen to fit with study results.

This has been addressed. The paragraph on pg 14, ln 318 now starts with: “Only recently, most U.S. institutions have created diversity, equity and inclusion (DEI) committees and task forces. GH programming should not be left out of DEI discussions and decisions.”

8- Line 299 not sure how appropriate it is to provide a web page since this was not a deliverable from this work. The authors could instead provide it as a reference so readers could check out the reference if they need the tool.

Thank you for your suggestion. The link has been removed.

9- Some reflection
Under bi directionality, the curriculum for the trainees from LMICs to US could be analyzed as well and then the 2 compared to understand which group (US trainees vs LMIC trainees) benefits most.

In the discussion and in looking at racism, the authors could make comment on the presence or lack of similar curricular in countries that were colonised. About directionality, the curricula sometimes encourages trainees to go teach at international sites with less emphasis on their need to learn from these communities. However, the partners come over to the US as learners and the idea that these can also teach something in the US is lacking. And whether the LMIC trainees can also conduct a research project in the US yet the US Trainees conduct research in LMICs.

Thank your for your valuable reflections and suggestions. These are all valid points for our author group to ponder and share with our colleagues and learners as US based GH educators. In keeping with an anti-colonial stance, we feel it would be out of place for us to comment on curricular contents of medical training programs in formerly colonized nations, especially since our group does not include anyone primarily practicing in a formerly colonized nation.

We echo your thoughts on the disparity of roles and scope of practice in bi-directional exchanges. Hudspeth et al. (2019) outline the systematic barriers to correcting some of these disparities in their paper.

10- Advise the author to outline their positionality and reflexivity in relation to this research. The author correctly identified a lack of representation and diversity in the Faculty of the GH curricula surveyed. What is the author’s positionality? In the methods section, the authors mentioned their ‘subject matter expertise’. What is their subject matter expertise? How is this expertise defined and assessed? Are LMIC partners involved in the assessment of US GH programs? Advise the authors to include a positionality and reflexivity statement.

Thank you for these insightful and important questions. Individual positionality statements and a group reflexivity statement has been added as an appendix.

Our author group is a varied group of physicians who have years of experience teaching global health to medical trainees in the USA, practicing medicine and building partnerships with medical professionals and institutions in communities affected by health inequities in the USA and LMICs. In addition, many of us have held or hold global health education leadership positions at the national level in the US. We draw our subject matter expertise from these experiences, and it can be assessed through some of the literature and work many of our authors have produced over the years (a non-exhaustive list of our work includes: Hudspeth et al., 2019; Lukolyo et al., 2016; Haq et al., 2022, McHenry et al, 2021; Steenhoff et al., 2017; Rule et al., 2022; Miller et al., 2022; Umphrey et al., 2024; St Clair et al., 2019; Rule et al., 2018). This group has also spent the past four years developing a curriculum titled “Global Health Education for Equity, Anti-Racism and Decolonization” GHEARD. While we all have more to learn on anti-racism and anti-colonialism, the extensive research and conversations we engaged in throughout this curriculum development process has tremendously contributed to our understanding of how racism and colonialism affect global health practice.

This study is part of a series of tasks undertook by the GHEARD working group. We regret not having authors from LMICs in this study. The timing of the study was particularly challenging as the survey was designed and distributed at the height of the COVID-19 pandemic when many of our LMIC partners were grappling with logistical challenges in their clinical work. The GHEARD curriculum working group that utilized the results of this study to develop GHEARD does include many partners from Indigenous nations and LMICs. They were aware of this survey, were present when the survey group asked for feedback and opted to focus their bandwidth on the curriculum development and implementation tasks over exploring the needs of the US programs in this survey.

11- The method by which the survey was developed is not entirely clear. In global health, we too often focus on the research that is conducted but not why it is conducted. Why were these particular characteristics chosen (pre-departure training, bidirectional partnerships, trainee evaluation)? How were they chosen? Which characteristics are not examined? All these aspects require more attention. It is good that the authors included pre-departure training of the host country’s culture, history, geopolitics, and language, history of colonialism, and etc. Cultural competence appears to be a glaring component of the training curricula that is missing. Another area that is lacking is impact assessment – assessment of the impact of trainee placements by LMIC institutions in terms of impact on the host facility, trainees, and patient care.

Thank you for these thoughtful comments. These particular characteristics were chosen based on prior literature and anecdotal reports that pre-departure training improves GH education (not necessarily research) experiences and exchanges and preparation in some of those areas are desired by international institutions that host US trainees (Cherniak et al., 2017; Eichbaum et al. 2015, Binagwaho et al., 2022, Kalbarczyk et al., 2019).

In this study, we prefer to use the term “cultural humility” (defined by Hook et al. (2013) as having an interpersonal stance that is other-oriented rather than self-focused, characterized by respect and lack of superiority toward an individual’s cultural background and experience) as opposed to “cultural competence”. Given the complex and evolutive nature of cultures, we do not believe that competence can ever be achieved in any culture, let alone one that is not one’s primary culture. In this study, 84% of survey participants reported that their program’s GH curriculum included teaching on cultural humility (pg 8, line 188).

While we agree with you that impact assessments are paramount to understanding the real value of GH trainee placements, we feel that they are out of the scope of this study which aimed to evaluate the strengths and weaknesses of global health curricula for medical trainees in the United States prior to placement experience. We feel that standardized learner evaluations could be a proxy for impact assessments if they are implemented systematically.

12- It would be better if characteristics surveyed were divided into different sections, e.g. knowledge of global inequities (hx of colonialism), knowledge of local context (history, culture, language), among others. Currently, the categories appear haphazardly selected and arranged. The survey design would benefit from better theoretical integration of decolonial and anti-racist theories.

Thank you for this suggestion, we have divided the curriculum content into the following sections: history of global health (history of colonialism, history of global health), history of manifest destiny (indigenous health, history of US racial disparities, current US racial health disparities, immigrant and refugee health, health inequity), systemic bias and intersectionality (anti-racism, white saviorism, critical consciousness, cultural humility, examination of motivation for GH engagement, social media, photography ethics), power dynamics in GH electives and partnerships. These sections are based on a comprehensive review of existing curricula, GH education and decolonization of global health education literature. This is expanded on in a separate paper (Fanny et al., 2024) by GHEARD authors. In that paper, we also expound on the importance of the integration of anti-racist, anti-colonialist pedagogy to address gaps. In addition, a GHEARD author also has mapped out decoloniality milestones for learners and that further informed these sections (Ratner et al., 2022).

We appreciate the reviewer suggestion of theoretical integration of anti-racist and decolonial frameworks into our survey and methodology. First, this survey represents a single task that grew out of a large multi-national workgroup of over 40 authors from 10 nations that came together as grassroots working group to explore and develop an anti-colonial and anti-racist curriculum for US based GH training programs. Second, an ARAC lens can mean a lot of things (Hunt and Riegelman, 2023). However, we did make efforts to decenter traditional topics in GH education by asking about ARAC topics. In addition, we attempted to use an iterative and participatory process with designing the survey. We did include the expertise and voices of a wide variety of experts including those in former colonial contexts in the iterative process of survey development. We describe more about this group in Fanny et al. (2024) and in another forthcoming paper that is under review (Barnes et al., 2023). We do briefly now allude to it on pg 5, ln 108 and we have added acknowledgements so this is further clarified. Ideally to further decenter whiteness and colonial power, we would like to have had a qualitative component to this study. That was part of the original plan. Using mixed methodology, we could have delved deeper into barriers to integration of anti-racism and anti-colonialism in US education curricula and pedagogies, as well as the barriers faced by URiM and IMG leaders in GH. This is described in the limitations section on pg 15, ln 355-358: *“Ideally, our team would have liked to add a qualitative component to further explore barriers to integration of anti-racism and decolonial curriculum and better describe IMG, URiM and LMIC perspectives on GH education. The pandemic and logistical challenges made that not possible but our team sees this as a key next step.“*

Finally, a key tenet of ARAC research practice is using data to empower and cultivate true change in communities (Hunt and Riegelman, 2023). Our survey team and the wider GHEARD working group used the results of this study and the literature review to create an open access, modular curriculum to integrate ARAC into US-based GH GME programs and beyond. This curriculum has already been piloted for over 300 learners in the US GME space and is endorsed by CUGH, AAP, APPD with the goal of creating true change in the field of GH.

13- The discussion is interesting and well-written. It should be better if the call to action or the implications of the study are more clearly highlighted. What is the change that the authors wish to see? What is the purpose of the study? The authors highlighted implications under each subheading. However, it will be nice if they could be tied together at the end. E.g. ‘Anti-racism and anticolonialism should be better integrated in US GH curricula at the institutional level. GH educational leadership needs to be diversified. GH partnerships should be bidirectional and have formal evaluation mechanisms. This will allow US GH programs to be an active form in anti-racism and decolonisation rather than perpetuating colonial inequities, etc’

Thank you for this suggestion. We have added it to the conclusion paragraph which now reads: “Anti-racism and anti-colonialism should be better integrated in US GH curricula and at the institutional level. GH educational leadership needs to be diversified. GH partnerships should be bidirectional and have formal evaluation mechanisms. We believe these changes will allow US GH programs to be active partners and allies in anti-racism and decolonization rather than perpetuating colonial inequities.”

14- Beyond having institutional policies on GH programs and short-term GH engagement. Are there other policy implications from this study? Are there standards that specialty training boards can reinforce for training program accreditation?

We agree with your suggestion that specialty training boards could also implement standards for training program accreditation and have included this point in the text which now reads: “Regulatory bodies such as specialty boards and the accreditation council for graduate medical education (ACGME) also have a potential role to play in the ARAC transformation of GH education. For example, they could include more rigorous standards such as ensuring LMIC partner participation in all aspects of decision-making and results sharing for research and trainee evaluation for clinical work conducted in a foreign setting as part of specialty training.”

15- Line 53. Delete the word ‘within’ in this sentence: ‘this study aimed to identify curricular strengths and gaps in ARAC content within for pediatric, family medicine and emergency medicine trainees participating in GH.’

This has been corrected.
